# Genome Sequences of Mycobacteriophages Luchador and Nerujay

**DOI:** 10.1128/genomeA.00599-15

**Published:** 2015-06-18

**Authors:** Welkin H. Pope, Taha Ahmed, Marissa K. Drobitch, David R. Early, Soukaina Eljamri, Naomi S. Kasturiarachi, Emily F. Klonicki, Daniel T. Manjooran, Aífe N. Ní Chochlain, Andrew O. Puglionesi, Vinod Rajakumar, Katherine A. Shindle, Mai T. Tran, Bryony R. Brown, Bryce M. Churilla, Karen L. Cohen, Kellyn E. Wilkes, Sarah R. Grubb, Marcie H. Warner, Charles A. Bowman, Daniel A. Russell, Graham F. Hatfull

**Affiliations:** Department of Biological Sciences, University of Pittsburgh, Pittsburgh, Pennsylvania, USA

## Abstract

Luchador and Nerujay are two newly isolated mycobacteriophages recovered from soil samples using *Mycobacterium smegmatis*. Their genomes are 53,387 bp and 53,455 bp long and have 96 and 97 predicted open reading frames, respectively. Nerujay is related to subcluster A1 phages, and Luchador represents a new subcluster, A14.

## GENOME ANNOUNCEMENT

Mycobacteriophages—viruses of mycobacterial hosts—form the largest collection of sequenced phages known to infect a common host, *Mycobacterium smegmatis* (1). They can be readily isolated from environmental samples and are genetically diverse, with different types grouped into 22 clusters (A to V) distinct from each other in both nucleotide sequence and gene content, and eight singletons that have no close relatives (1, 2). The largest is cluster A containing over 300 individual members (http://phagesdb.org), although these span considerable genetic diversity and can be grouped into 13 subclusters (A1 to A13) according to their nucleotide sequence similarity. Some members of subclusters A2 and A3 have been shown to efficiently infect *M. tuberculosis*, whereas members of subclusters A1, A4, A5, A6, A7, A8, and A9 do not (3).

Mycobacteriophages Luchador and Nerujay were isolated from soil samples collected in Pittsburgh, PA; Luchador by direct plating of the sample, and Nerujay following enrichment with *M. smegmatis* mc^2^155. Electron microscopy shows that both have siphoviral morphologies with isometric heads. Following purification and amplification, DNA was isolated from both phages and sequenced on an Illumina MiSeq using 140-bp single-end reads. Reads from each phage were assembled using Newbler, each into a major contig with 1,198-fold and 300-fold coverage for Luchador and Nerujay, respectively. Both phages have 10 base 3′ extensions at their ends but with different sequences; for Luchador, 5′-CGGTCGGTTA, and for Nerujay, 5′-CGGATGGTAA. The genome lengths are 53,387 bp and 53,455 bp, respectively and their G+C contents are 62.1% and 63.7%. Annotation of the genomes identified 96 and 97 protein coding genes in Luchador and Nerujay, respectively, and Luchador encodes a single tRNA (tRNA^asn^).

Both Luchador and Nerujay have extensive nucleotide sequence similarity to other cluster A mycobacteriophages. The closest relatives of Nerujay are Aeneas and PhrostyMug and it is easily grouped with these in cluster A1. The closest relative of Luchador is Pukovnik, a subcluster A2 phage. However, the genomes share 80% nucleotide identity but this spans only 45% of the genome lengths, and we propose that Luchador is the founding member of a new subcluster, A14. We also note that a relatively high proportion of Luchador open reading frames (ORFs) (9 of 97) are orphans (genes with no homologues in other mycobacteriophages), a characteristic feature of single-genome subclusters.

Both Luchador and Nerujay form turbid plaques and encode a predicted repressor protein (Luchador gp78 and Nerujay gp79) consistent with temperate lifestyles. Nerujay encodes an integrase of the serine recombinase family, whereas Luchador has a partitioning system that includes *parA* and *parB* genes, as seen in some other cluster A mycobacteriophages (3, 4). Nerujay also contains multiple copies of a putative stoperator sequence related to the consensus sequence reported for Bxb1 (5). Luchador also has multiple putative stoperator sites, but they conform to a consensus that is different from that in other cluster A phages, consistent with its designation within the new subcluster A14. The Luchador tRNA^asn^ is distinct in sequence from those encoded by all other cluster A phages.

### Nucleotide sequence accession numbers. 

The Luchador and Nerujay genome sequences are available from GenBank under the accession numbers KR080193 and KR080201, respectively.
